# Paeoniflorin alleviates liver injury in hypercholesterolemic rats through the ROCK/AMPK pathway

**DOI:** 10.3389/fphar.2022.968717

**Published:** 2022-08-23

**Authors:** Tong Liu, Ning Zhang, Lingya Kong, Sijie Chu, Ting Zhang, Guangdi Yan, Donglai Ma, Jun Dai, Zhihong Ma

**Affiliations:** ^1^ School of Basic Medicine, Hebei University of Chinese Medicine, Shijiazhuang, Hebei, China; ^2^ Department of Infectious Disease, The Third Hospital of Hebei Medical University, Shijiazhuang, Hebei, China; ^3^ Experimental Center, Hebei University of Chinese Medicine, Shijiazhuang, Hebei, China; ^4^ School of Pharmacy, Hebei University of Chinese Medicine, Shijiazhuang, Hebei, China; ^5^ Hebei Key Laboratory of Integrative Medicine on Liver-Kidney Patterns, Shijiazhuang, Hebei, China

**Keywords:** paeoniflorin, rho kinase (ROCK), AMPK, anti-inflammation, anti-oxidation, hypercholesterolemia

## Abstract

Paeoniflorin (PF) is the main active component in *Paeonia lactiflora Pall*, and it has multiple effects. However, the precise mechanism of PF in hypercholesterolemia is unclear. In this study, rats were either fed a high-cholesterol diet (HCD) for 4 weeks to establish the hypercholesterolemic model or administered normal saline or PF (20 mg/kg/day). PF significantly reduced liver weight and the liver index. PF reduced hepatic lipid deposition and inflammation, improved serum lipid metabolism, and significantly inhibited serum and hepatic oxidative stress and the inflammatory response. PF treatment caused a marked decrease in the phosphorylated myosin phosphatase target subunit (p-MYPT)-1, nuclear sterol regulatory element-binding protein-1c (SREBP-1c), fatty acid synthase (FAS) levels, and an increase in the low-density lipoprotein receptor (LDLR) and phosphorylated-AMP-activated protein kinase (p-AMPK). Thus, PF could alleviate liver injury in hypercholesterolemic rats, and the specific mechanism may be related to the antioxidant, anti-inflammatory properties, and ROCK/AMPK/SREBP-1c signaling pathway.

## Introduction

Hypercholesterolemia is a lipid metabolic disorder characterized by an increase in blood cholesterol, particularly low-density lipoprotein cholesterol (LDL) (Csonka et al., 2016). Epidemiological studies indicated that dietary habits such as intake of foods containing excessive saturated fats and cholesterol are the risk factors for hypercholesterolemia (AlSaad A. M. et al., 2020). Hypercholesterolemia is an important cause of obesity, atherosclerosis, and nonalcoholic fatty liver disease (NAFLD). In 2015, the World Health Organization reported that approximately 4.5% of global deaths worldwide were due to hypercholesterolemia. Thus, it is significant to find effective drugs to treat hypercholesterolemia.

The pathogenesis of hypercholesterolemia remains unclear, and oxidative stress and the inflammatory response are often mentioned as key factors (Zhang et al., 2022). Studies have shown that excessive accumulation of cholesterol in the blood leads to increased reactive oxygen species (ROS) production (Li et al., 2020), and these highly reactive species mediate low-grade inflammation (Reyes-Gordillo et al., 2017) (Liao et al., 2021) (Incalza et al., 2018). Systematic oxidative stress and inflammatory response impair lipid metabolism, and thus, lipids accumulate in the liver (Abbasi et al., 2021). Therefore, reducing the oxidative stress and the inflammatory response is an important strategy to reduce hepatic lipid deposition and protect the liver in hypercholesterolemic rats.

An increasing amount of evidence showed that Rho kinase (ROCK) is closely related to oxidative stress and the inflammatory response (Zhang et al., 2019; Matoba et al., 2020). Fasudil, a ROCK inhibitor, inhibits ROCK activation and reduces oxidative stress and the inflammatory response in hypercholesterolemic rats (Ma et al., 2011a; Ma et al., 2011b). A recent study reported that hepatic ROCK suppresses AMP-activated protein kinase (AMPK) activity, and the ROCK/AMPK pathway is required to mediate hepatic lipogenesis during overnutrition (Huang et al., 2018). A large amount of evidence showed that activating the AMPK pathway can improve lipid deposition through the key downstream pathway in NAFLD (Xiao et al., 2019; Huang et al., 2021). Sterol regulatory element-binding protein-1c (SREBP-1c) is downstream of the AMPK pathway and plays an important role in gene expression related to intracellular lipid synthesis such as fatty acid synthase (FAS) (Mohammadi et al., 2020). At the same time, the activation of AMPK can increase the level of low-density lipoprotein receptor (LDLR) protein to regulate cholesterol homeostasis (Li et al., 2021). Taken together, ROCK inhibition and AMPK activation are potential targets to reduce oxidative stress, the inflammatory response, and hepatic lipid deposition in hypercholesterolemia.

Paeoniflorin (PF) has well known for pharmacological effects such as anti-inflammatory, anti-oxidation, reduces lipid deposition (Ma et al., 2020). Our previous studies showed that PF could reduce oxidative stress and the inflammatory response and improve hepatic lipid metabolism in NAFLD rats induced by high-fat diet (HFD) (Ma et al., 2017). However, the beneficial effects of PF on liver damage induced by hypercholesterolemia and the potential molecular mechanism remain unclear. We investigated whether PF reduces oxidative stress and inflammation response and improves lipid metabolism by inhibiting ROCK activation, and in turn, influencing the AMPK/SREBP-1c/FAS pathway. In this study, we established a hypercholesterolemic rat model using a high-cholesterol diet (HCD) to explore the hepatic protection effect and potential mechanism of PF in hypercholesterolemia.

## Materials and methods

### Materials

**FIGURE 1:**
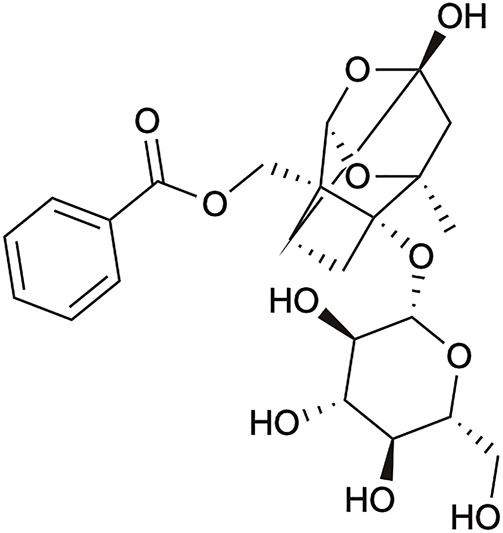
The general structure of paeoniflorin.

PF (purity 98%) (Figure 1) was purchased from Nanjing Zelang Pharmaceutical Technology Co., Ltd. (Nanjing, China). Cholesterol was purchased from Beijing Solarbio Science and Technology Co., Ltd (Beijing, China). The normal diet and HCD were provided and supervised by Hebei Medical University (Shijiazhuang, China). Myosin phosphatase target subunit (MYPT)-1 and phosphorylated MYPT (p-MYPT)-1 primary antibodies were purchased from Affinity Biosciences Ltd. (Jiangsu, China), AMPK and phosphorylated-AMPK antibodies were purchased from Shanghai Abways Biotechnology Co., Ltd. (Shanghai, China), LDLR, SREBP-1c, and FAS antibodies were purchased from Jiangsu Affinity Biosciences Co., Ltd (Jiangsu, China), glyceraldehyde 3-phosphate (GAPDH) antibodies were purchased from Bioworld Technology, lnc. (Nanjing, China). Secondary horseradish peroxidase (HRP) antibodies were purchased from Shanghai Abways Biotechnology Co., Ltd. (Shanghai, China).

### Animals and experimental design

Forty specific pathogen-free male Sprague–Dawley (SD) rats, weighing 180–200g, were provided by Hebei Medical University (Shijiazhuang, China). All rats were housed in cages under standard conditions (e.g., kept at room temperature, 24 ± 2°C) with free access to food and water. All animal handling procedures were performed in accordance with the 1996 National Institutes of Health Guide for the Care and Use of Laboratory Animals and approved by the Ethics Committee for Animal Experiments at the Hebei University of Chinese Medicine (approval number: DWLL2020008).

Forty rats were randomly allocated to four groups (n = 10 per group), as follows: normal control group (NC), PF-treated control group (NPF), model group (MOD), and PF-treated model group (MPF). The NC group and NPF group were fed with normal diet, MOD group and MPF group were fed with HCD (HCD composition: protein 18%, fat 4%, fiber 5%, ash 8%, water 10%, cholesterol 2%, cholic acid 0.5%, total energy 1780 kcal/kg) for 4 weeks. Within 4 weeks of HCD feeding, both NPF group and MPF group were administered PF (20 mg/kg/day) orally (Ma et al., 2017), and the NC and the MOD groups were administered the same volume of normal saline orally. Food intake was monitored daily and body weight was measured weekly during the experiment. After 4 weeks, rats were fasted for 12 h, weighed, and then intraperitoneally injected with pentobarbital (40 mg/kg) for anesthesia. Blood was collected from the femoral artery and the serum was immediately centrifuged for separation. The liver appearance was observed, quickly removed, weighed, and the liver index was calculated (liver weight/body weight × 100%). The same part of the liver was fixed in 4% paraformaldehyde, while the rest of the liver was temporarily stored in liquid nitrogen and stored in a −80 °C freezer until subsequent experimentation.

### Preparation of liver homogenate

Liver tissue from each group was accurately weighed and ground in a homogenate medium. After centrifugation at 2,500 rpm for 10 min, the supernatant was removed for analysis.

### Detection of biochemical indicators in serum and liver

According to the kit instructions, serum and liver homogenates were used to detect total cholesterol (TC), triglycerides (TG), LDL, and high-density lipoprotein cholesterol (HDL) levels. Serum aspartate aminotransferase (AST) and alanine aminotransferase (ALT) activity were also detected. The above kits were purchased from Nanjing Jiancheng Bioengineering Institute Co., Ltd. (Nanjing, China).

### Detection of inflammation indicators in serum and liver

Enzyme-linked immunosorbent assays were used to detect serum and hepatic C-reactive protein (CRP), tumor necrosis factor (TNF)-α, interleukin (IL)-1β, and IL-6 levels in serum and liver according to the kit instructions. The above kits were purchased from Hangzhou Lianke Biotechnology Co., Ltd (Hangzhou, China).

### Histological changes in the liver

From each rat, the same part of the liver tissue was fixed in 4% paraformaldehyde for over 48 h. The liver sample was dehydrated in gradient alcohol, embedded in paraffin wax, and then sliced into 4-μm thick sections and stained with hematoxylin-eosin (H and E) for observation. Each section was observed with a microscope at ×200 magnification (Leica DM4000B, Solms, Germany).

The frozen liver tissue was sliced into 8-μm thick sections using a cryomicrotome (Thermo Fisher Scientific Inc, Massachusetts, United States), fixed in fixing solution (Servicebio Technology Co., Ltd, Wuhan, China) for 15 min, and stained with oil red O solution (Servicebio Technology Co., Ltd, Wuhan, China) for 15 min, The liver tissues were washed with 60% isopropanol, and then the nuclei were then stained with hematoxylin staining solution (Servicebio Technology Co., Ltd, Wuhan, China). The slices were observed, and photos were taken using an optical microscope (Olympus, Tokyo, Japan).

### Detection of oxidative stress indicators in serum and liver

According to the kit instructions, the prepared serum and liver homogenates were used to detect superoxide dismutase (SOD) and catalase (CAT) activity and the malondialdehyde (MDA) and glutathione (GSH) content. The above kits were purchased from Nanjing Jiancheng Bioengineering Institute Co., Ltd. (Nanjing, China).

We used a dihydroethidium (DHE) probe to measure the ROS fluorescence intensity in the liver tissues in each group. Frozen fresh liver tissue was taken and sliced under freezing conditions for ROS detection. The frozen section was rewarmed at room temperature to control the water content. Spontaneous fluorescence quenching reagent (Servicebio Technology Co. Ltd., Wuhan, China) was added and incubated for 5 min followed by rinsing with running water for 10 min. ROS staining solution (Sigma, St. Louis, MO, United States) was added and incubated in a light-proof incubator at 37°C for 30 min. After the slides were cleaned with PBS, DAPI solution was added and incubated at room temperature without light for 10 min. The liver tissue slices were washed again with PBS, dried, and sealed with an anti-fade mounting medium. The sections were observed under a fluorescence microscope (Nikon eclipse C1, Nikon, Japan), and the images were collected.

### Western blot analysis of protein expression in liver

The frozen tissue was removed, and RIPA lysate (Servicebio Technology Co., Ltd, Wuhan, China), protease inhibitor, and phosphorylated protease inhibitor (Boster Biological Technology Co., Ltd. Wuhan, China) were added, and the tissue was homogenated. After centrifugation, the supernatant was extracted to prepare protein samples. Nuclear protein was extracted according to the instructions of the kit (Beyotime Biotechnology Co., Ltd. Shanghai, China) and used to detect the expression of nuclear SREBP-1c. The equivalent protein sample or nuclear protein sample was added to 8% sodium dodecyl sulfate-polyacrylamide gel to undergo electrophoresis, and the protein was then transferred to the polyvinylidene difluoride (PVDF) membranes. After blocking with 5% skimmed milk powder, the PVDF membrane was incubated overnight with the following antibodies: p-MYPT-1, MYPT-1, p-AMPK, AMPK, LDLR, SREBP-1c, FAS, or GAPDH at 4 °C. After cleaning the primary antibody, the membranes were incubated with the corresponding secondary antibody and then treated with the Super ECL detection reagent (Yeasen Biotechnology Co., Ltd. Shanghai, China). The obtained bands were analyzed using Vision Capt software (Kunming Beijie Technology Co., Ltd. Kunming, China).

### Statistical analysis

Results are presented as the mean ± standard error of the mean (S.E.M.). Data analysis was conducted using the GraphPad Prism 8.0.1 software package for Windows (GraphPad Software, America). We first tested the data’s normality and homogeneity of variance. The differences among the four groups were then estimated using a one-way analysis of variance followed by Tukey’s post-hoc test. *p* < 0.05 was considered to be statistically significant.

## Results

### Effect of PF on body weight, and food intake

**FIGURE 2:**
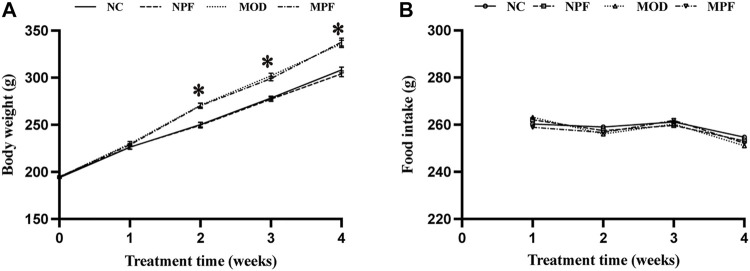
Effects of paeoniflorin (PF) on body weight, and food intake. **(A)** Weight changes for rats in the four groups during the treatment with PF. **(B)** Food intake of each rat in the four groups during the treatment with PF. The liver samples were obtained from the normal control group (NC), PF-treated control group (NPF), model group (MOD), and PF-treated model group (MPF). Data are presented as the mean ± S.E.M (n = 10). *p < 0.05 vs. NC and #*p* < 0.05 vs. MOD.

As shown in Figure 2, the weight of rats in the MOD group was significantly higher than that in the NC group (*p* < 0.05). There was no obvious difference in body weight between the MOD and MPF groups or between the NPF and NC groups, indicating that PF had no significant influence on the weight of the rats. Additionally, food intake did not change significantly during the treatment. Therefore, we excluded the anorexia effect of PF on hypercholesterolemia.

### Effects of PF on lipids level in serum

**FIGURE 3:**
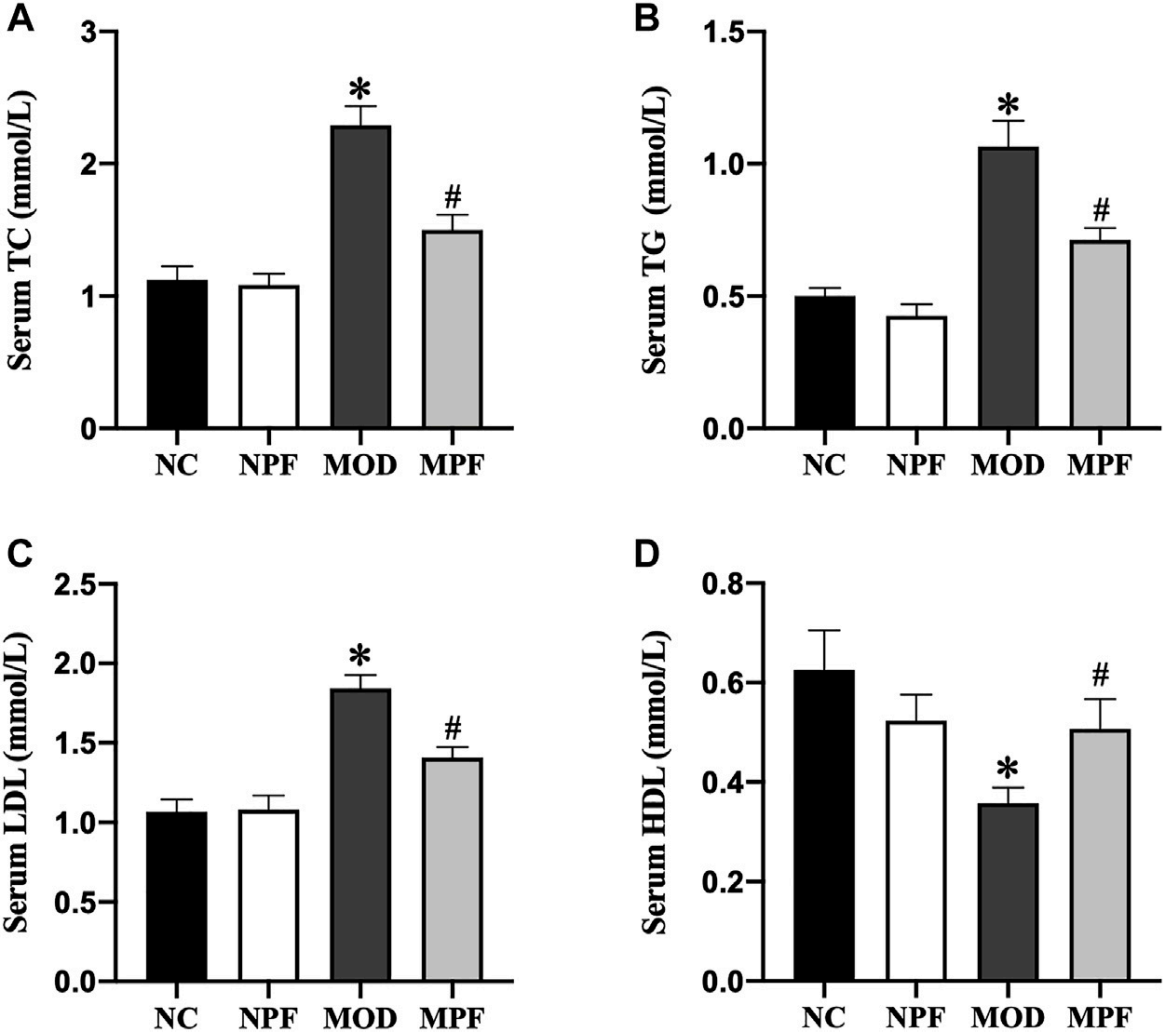
Effects of paeoniflorin (PF) on lipids level in serum. Serum levels of **(A)** TC, **(B)** TG, **(C)** LDL, and **(D)** HDL. Serum samples were obtained from the normal control group (NC), PF-treated control group (NPF), model group (MOD), and PF-treated model group (MPF). Data are presented as the mean ± S.E.M (n = 10). *p < 0.05 vs. NC and #*p* < 0.05 vs. MOD.

Serum lipid levels are presented in Figure 3. In the MOD group, the serum levels of TC, TG, and LDL were markedly higher than those in the NC group, and the HDL level was markedly lower than those in the NC group (all *p* < 0.05). After PF treatment, the serum TC, TG, and LDL levels were markedly decreased, and the serum HDL level was significantly increased (all *p* < 0.05). Among the above indicators, there were no differences between the NC and NPF groups.

### Effects of PF on oxidative stress indicators in the serum and liver

**FIGURE 4:**
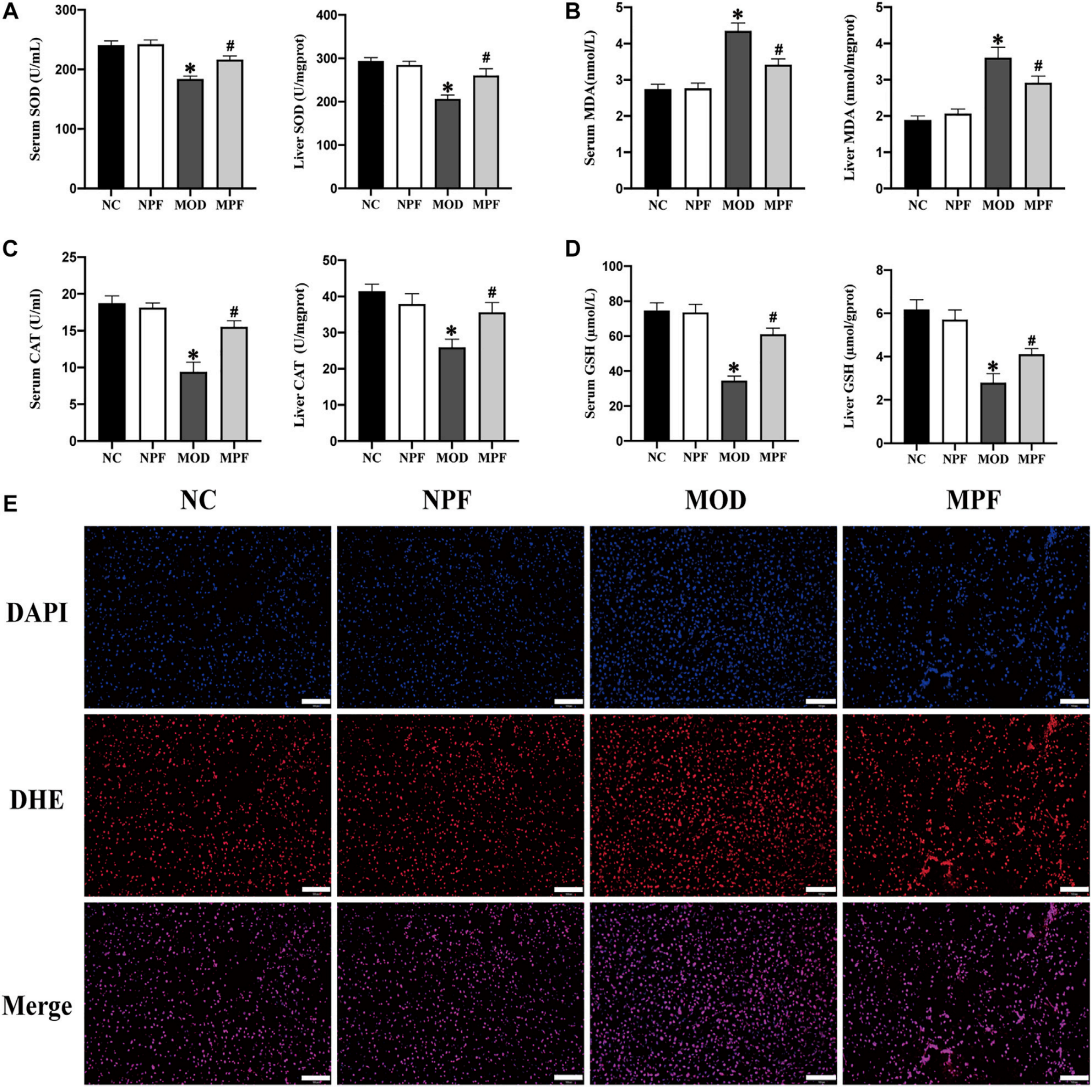
Effects of paeoniflorin (PF) on oxidative stress indicators in the serum and liver. **(A)** Serum and liver SOD activity. **(B)** Serum and liver MDA content. **(C)** Serum and liver CAT activity. **(D)** Serum and liver GSH content. **(E)** Representative reactive oxygen species (ROS) histologic slices in four groups, (200×, scale bar = 100 μm). The serum and liver samples were obtained from the normal control group (NC), PF-treated control group (NPF), model group (MOD), and PF-treated model group (MPF). Data are presented as the mean ± S.E.M (*n* = 10). *p < 0.05 VS. NC and #*p* < 0.05 VS. MOD.

Compared with the NC group, SOD and CAT activity and the GSH content in the serum and liver in the MOD group showed an obvious decrease. The serum and liver MDA content also increased significantly (all *p* < 0.05). PF treatment could increase SOD and CAT activity and the GSH content and decrease the MDA content (all *p* < 0.05) (Figures 4A–D). Compared to the NC group and the NPF group, there were no apparent differences.

For liver ROS sections, we used the DHE probe to detect ROS expression in liver tissue. Compared with the CON group, the MOD group had a higher fluorescence intensity. However, after PF treatment, the fluorescence intensity decreased significantly, which showed that PF could reduce ROS production in the liver (Figure 4E).

### Effects of PF on inflammatory cytokines in the serum and liver

**FIGURE 5:**
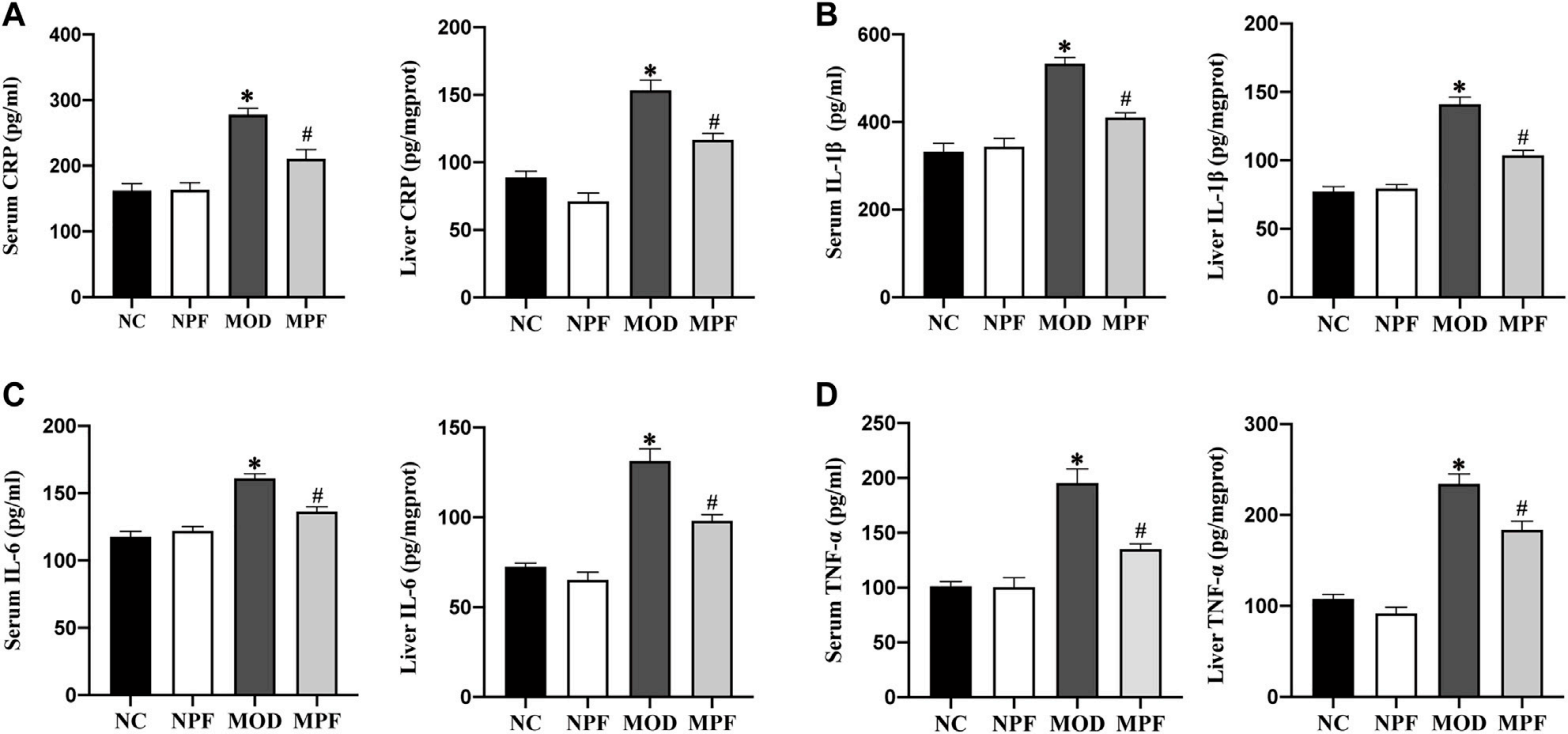
Effects of paeoniflorin (PF) on inflammatory cytokines in the serum and liver. Serum and liver levels of **(A)** CRP, **(B)** IL-1β, **(C)** IL-6, and **(D)** TNF-α. The serum and liver samples were obtained from the normal control group (NC), PF-treated control group (NPF), model group (MOD), and PF-treated model group (MPF). Data are presented as the mean ± S.E.M (*n* = 10). *p < 0.05 vs. NC and #*p* < 0.05 vs. MOD.

Serum and liver CRP, IL-1β, IL-6, and TNF-α levels in the MOD group were considerably higher than those of the NC and NPF groups (all *p* < 0.05). After PF treatment, these levels were all distinctly decreased (all *p* < 0.05) (Figure 5). There was no statistically significant difference between the NC and NPF groups.

### Effects of PF on liver function and histological changes in the liver

**FIGURE 6:**
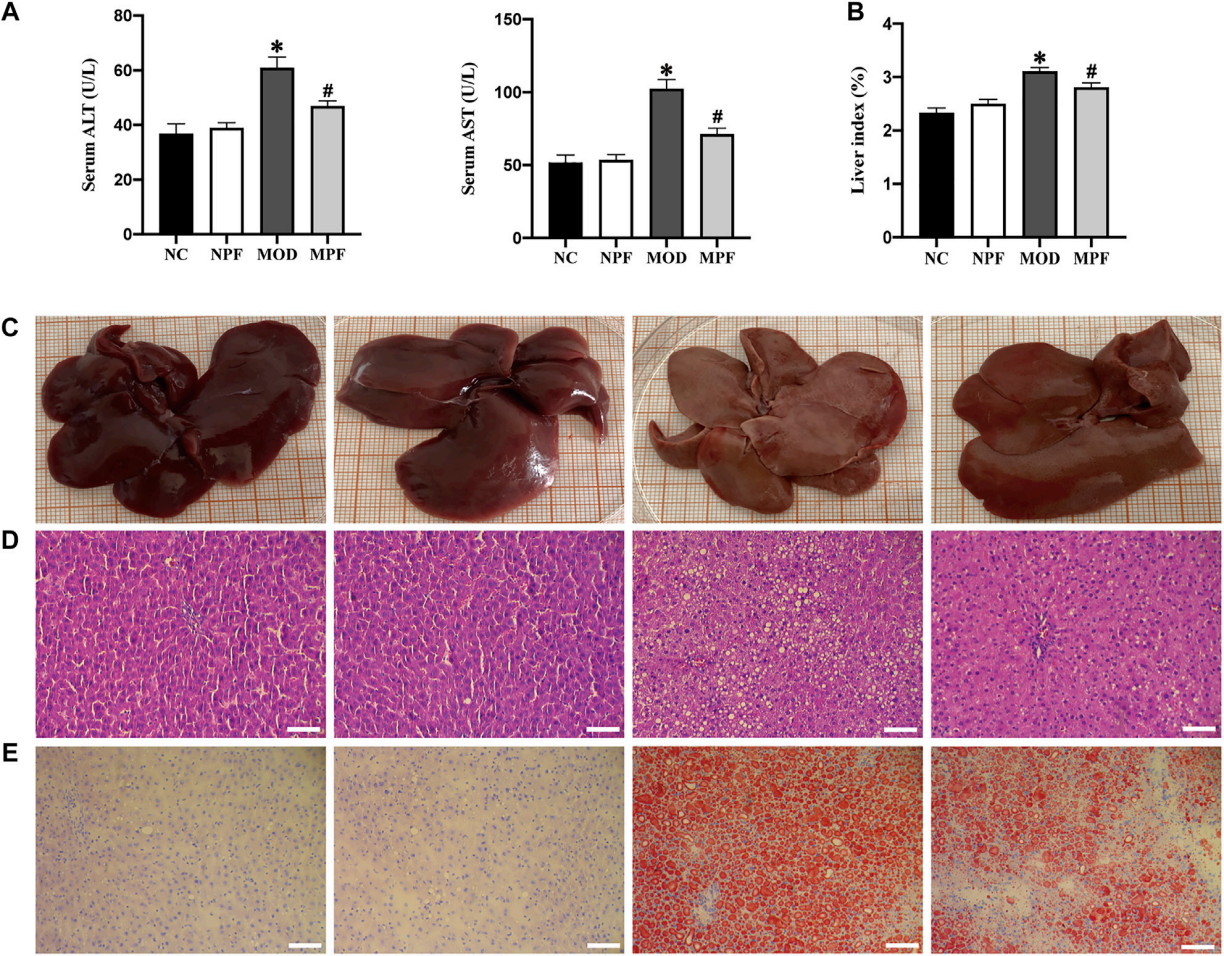
Effects of PF on liver function and histological changes in the liver. **(A)** Serum levels of ALT and AST activity. **(B)** Average liver index of rats in four groups. **(C)** Representative photos of rat liver in four groups. **(D)** Representative H and E staining histologic slices in four groups, (200×, scale bar = 100 μm). **(E)** Representative oil red O staining histologic slices in four groups, (200×, scale bar = 100 μm). The serum and liver samples were obtained from the normal control group (NC), PF-treated control group (NPF), model group (MOD), and PF-treated model group (MPF). Data are presented as the mean ± S.E.M (*n* = 10). *p < 0.05 vs. NC and #*p* < 0.05 vs. MOD.

Serum ALT, AST, and the liver index are important indicators reflecting liver injury. As shown in Figure 6A, compared with the NC group, ALT and AST activity showed an obvious increase in the MOD group. Conversely, the MPF group showed lower of ALT and AST activity (both *p* < 0.05). Compared with the NC group, the liver index in the MOD group was significantly higher. PF treatment also significantly reduced the liver index (*p* < 0.05) (Figure 6B).

Macroscopic observation showed that the livers of rats in the NC and NPF groups were chestnut-colored, while livers in MOD group rats were yellowish, butyrous, brittle, and larger than those in the NC and NPF groups. The livers of rats treated with PF had less histological damage than those of the MOD group (Figure 6C).

Microscopically, H&E staining of liver tissue showed that the NC and NPF group liver tissue had a normal structure with hepatocytes showing healthy nuclei and parenchymal structure, but the rats of the MOD group showed obvious fatty changes and inflammatory cell infiltration in liver sections. After PF treatment, liver lipid deposition and inflammatory cell infiltration were significantly reduced (Figure 6D).

To evaluate the effect of PF on liver lipid deposition, we used oil red O staining to detect the lipid deposition in the livers of each group. In Figure 3B, compared to the NC group, lipid deposition was significantly increased in the MOD group. After PF treatment, lipid deposition was significantly reduced in the MPF group, indicating that PF could reduce liver lipid deposition (Figure 6E).

### Effects of PF on lipids level in liver

**FIGURE 7:**
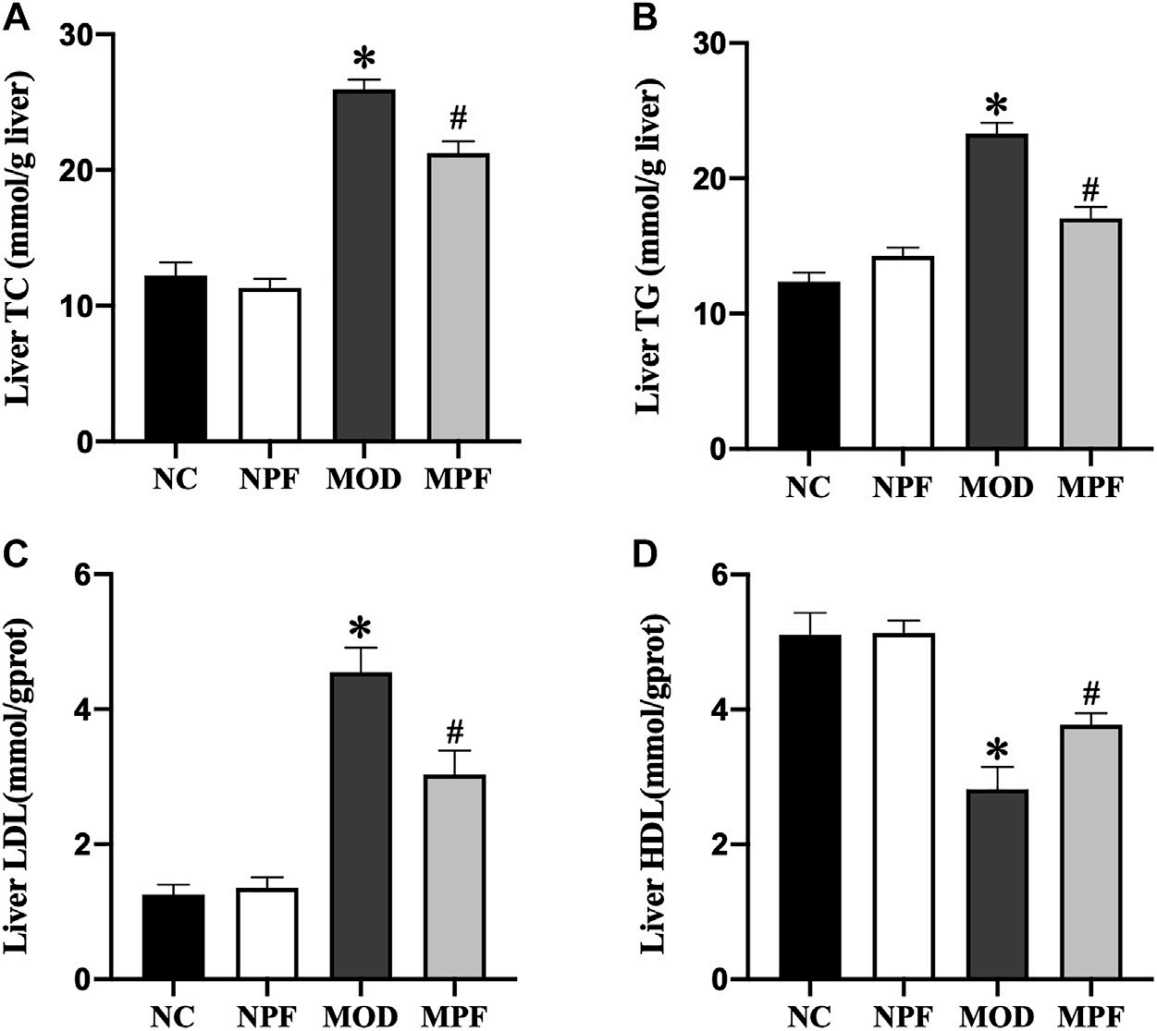
Effects of paeoniflorin (PF) on lipids in the liver. Hepatic levels of **(A)** TC, **(B)** TG, **(C)** LDL, and **(D)** HDL. The liver samples were obtained from the normal control group (NC), PF-treated control group (NPF), model group (MOD), and PF-treated model group (MPF). Data are presented as the mean ± S.E.M (n = 10). *p < 0.05 vs. NC and #*p* < 0.05 vs. MOD.

As shown in Figure 7, in the MOD group, the liver levels of TC, TG, and LDL were markedly higher than those in the NC group, and the HDL level was markedly lower than those in the NC group (all *p* < 0.05). The liver TC, TG, and LDL levels were markedly decreased after PF treatment, and the liver HDL level was significantly increased (all *p* < 0.05). Among the above indicators, there were no differences between the NC and NPF groups.

### Effects of PF on protein expression related to lipid metabolism in the liver

**FIGURE 8:**
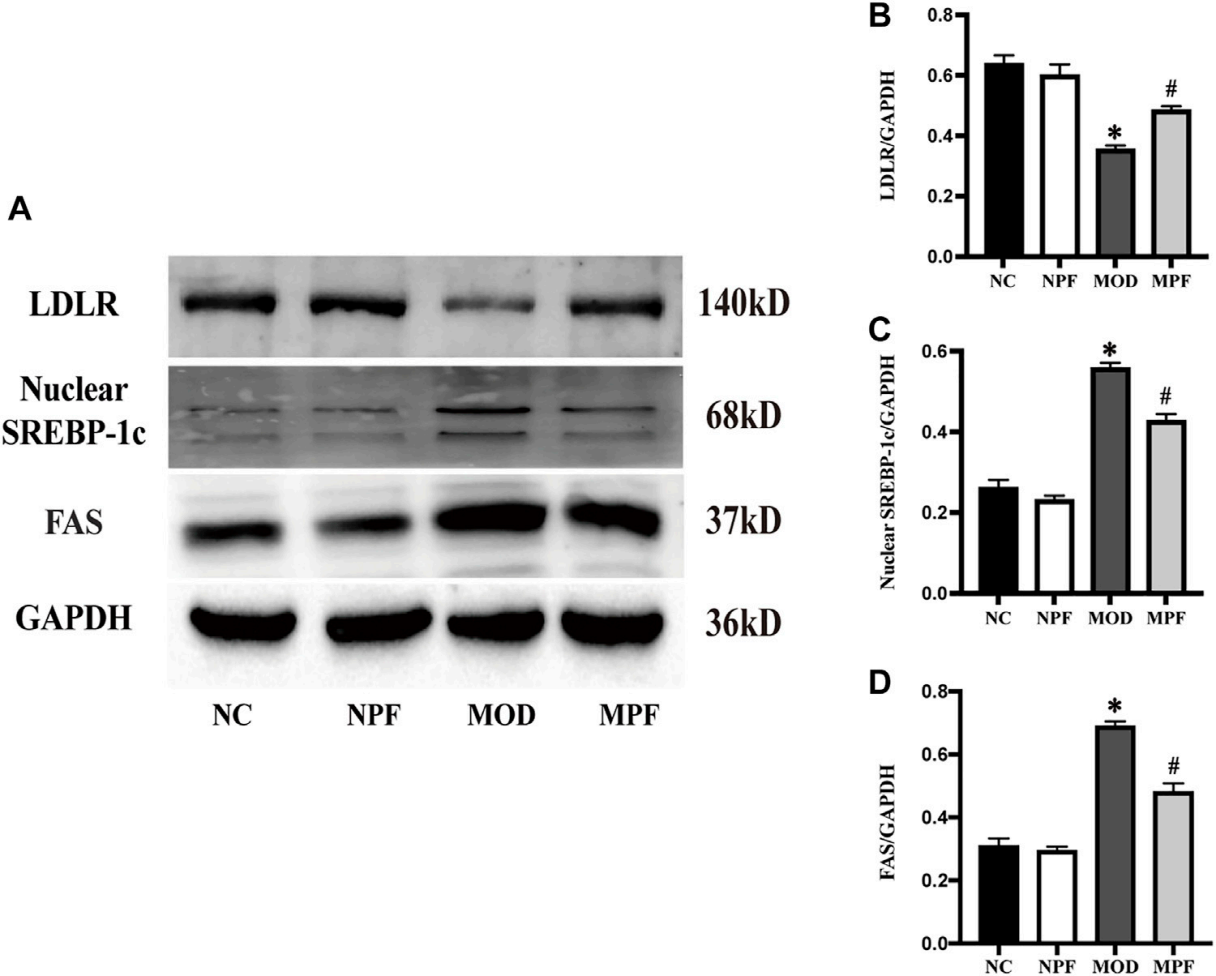
Effects of paeoniflorin (PF) on protein expression related to lipid metabolism in the liver. **(A)** Representative LDLR, nuclear SREBP-1c, and FAS expression brands. **(B–D)** Grey value of western blotting bands. The liver samples were obtained from the normal control group (NC), PF-treated control group (NPF), model group (MOD), and PF-treated model group (MPF). Data are presented as the mean ± S.E.M (n = 3). *p < 0.05 vs. NC, #*p* < 0.05 vs. MOD.

In Figure 8, LDLR protein expression in the MOD group was down-regulated remarkably compared to the NC group. After PF treatment, LDLR protein expression was significantly increased (*p* < 0.05). FAS and nuclear SREBP-1c protein expression in the MOD group was considerably higher than in the NC group. After treatment with PF, FAS and nuclear SREBP-1c protein expression was down-regulated rapidly (*p* < 0.05). There was no statistically significant difference between the NC and NPF groups.

### Effects of PF on p-MYPT-1/p-AMPK protein expression in the liver

**FIGURE 9:**
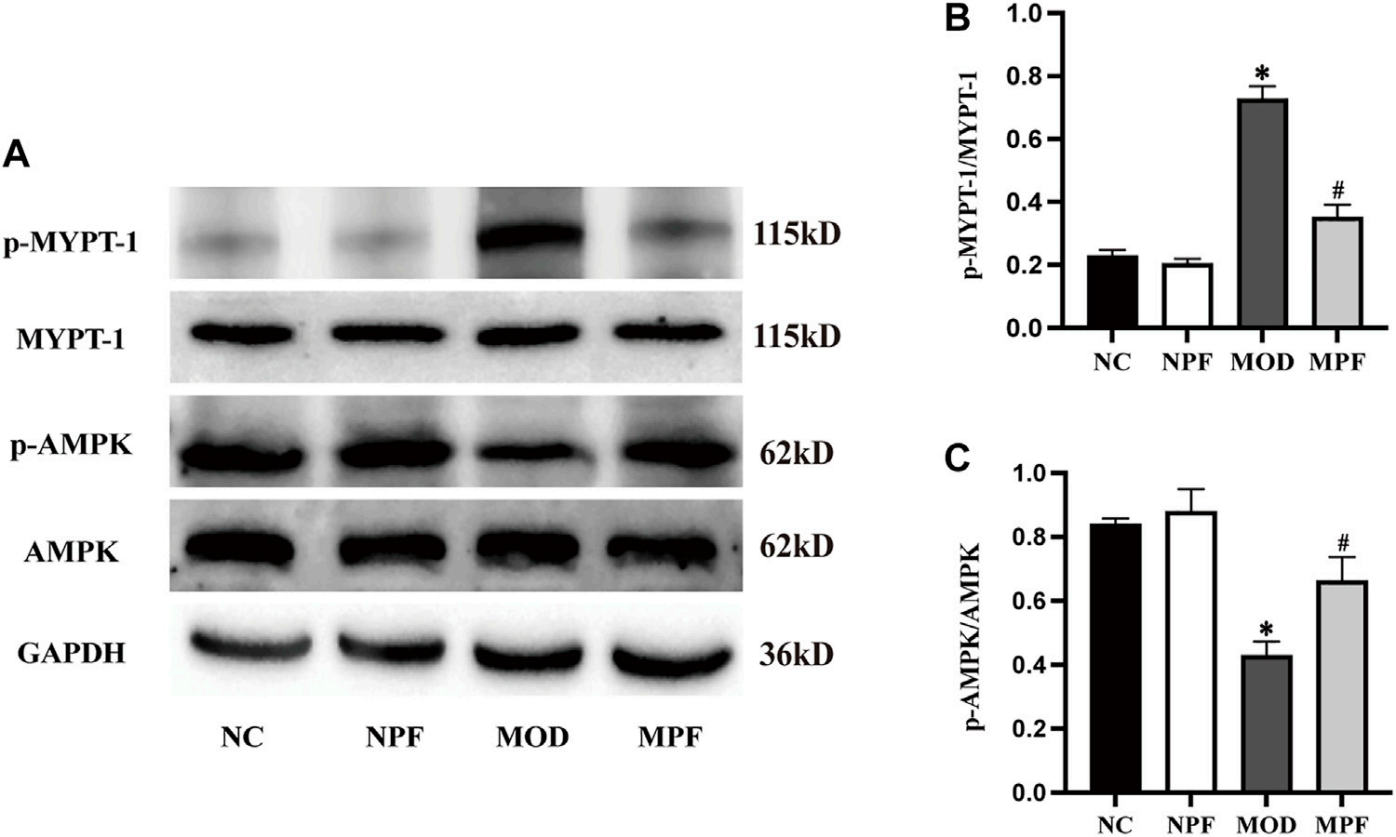
Effects of paeoniflorin (PF) on p-MYPT-1/p-AMPK protein expression in the liver. **(A)** Representative p-MYPT-1 and p-AMPK expression bands. **(B,C)** Grey value of western blotting bands. The liver samples were obtained from the normal control group (NC), PF-treated control group (NPF), model group (MOD), and PF-treated model group (MPF). Data are presented as the mean ± S.E.M (*n* = 3). *p < 0.05 vs. NC, #*p* < 0.05 vs. MOD.

As shown in Figure 9, p-AMPK protein expression in the MOD group was down-regulated remarkably compared to the NC group. However, after treatment with PF, p-AMPK expression was significantly up-regulated (*p* < 0.05). Compared with the NC and NPF group, the MOD group rats had higher liver p-MYPT-1 protein expression (*p* < 0.05), and PF treatment decreased its expression in liver tissue (*p* < 0.05). There was no statistically significant difference between the NC and NPF groups.

## Discussion

At present, hypercholesterolemia is mainly treated by lifestyle intervention and drugs. Therapeutic lifestyle interventions include diet control, increased exercise, smoking cessation, and alcohol restriction, etc. Drugs mainly include statins, intestinal cholesterol absorption inhibitors, bile acid chelators and other lipid-lowering drugs. Long term use of these drugs often has hepatotoxicity and gastrointestinal reactions. PF is the main active component of *Paeonia Lactiflora Pall* and has the effect of hepatic protection, anti-inflammation, anti-oxidation, and lipid metabolism. In our previous study, we used HFD feeding for 14 weeks to establish NAFLD rat model and evaluate the insulin-sensitizing effect of PF and possible molecular mechanisms. However, the beneficial effects of PF on hypercholesterolemia-induced liver injury and the underlying molecular mechanisms remain unclear. In this study, we used the HCD for 4 weeks to establish the hypercholesterolemic model, and we explored the mechanism by which PF attenuated the development of hypercholesterolemia in rats. PF could inhibit ROCK, activate AMPK, and then inhibit the downstream SREBP-1c, FAS and LDLR to play the role of antioxidant, and anti-inflammatory, reducing lipid deposition and protecting the liver.

Because dietary intake of cholesterol is a critical factor in the etiopathogenesis of hypercholesterolemia, it is common for the HCD to induce hypercholesterolemia in rats (Cunha et al., 2021). It was shown that rats fed a HCD for 4 weeks could be used as an experimental animal model for hypercholesterolemia, and these rats showed a significant increase in blood cholesterol, especially LDL (Fidèle et al., 2017). This showed that we were successful in establishing the model for hypercholesterolemia in rats (AlSaad A. M. S. et al., 2020). HCD can increase the serum lipid level, while reducing the lipid level can alleviate hypercholesterolemia progression. We also detected the levels of other lipids in serum, including TC, TG, and HDL, and PF decreased serum TC, TG, LDL, and HDL in hypercholesterolemic rats. LDLR is widely expressed and is a key receptor for maintaining cholesterol homeostasis. It has been confirmed that the majority of blood LDL-C is recognized and eliminated by the endocytic cycle of LDLR in hepatocytes (Go and Mani, 2012; Yang et al., 2020). We found that PF could up-regulate the expression of LDLR, which may be one of the mechanisms of PF reducing serum cholesterol level. The specific mechanisms of PF to reduce blood lipids may be related to 3-hydroxy-3-methyl glutaryl coenzyme A reductase (HMG-CoA) and proliferator-activated receptor-alpha (PPAR-α). This is consistent with the results of Hu et al. (Hu et al., 2017).

Hypercholesterolemia can cause oxidative stress and inflammation. Excessive accumulation of cholesterol in the blood can cause significant ROS accumulation and affect the redox imbalance in tissues (Cammisotto et al., 2021). When the balance between the production of free radicals and endogenous antioxidants is destroyed, ROS is accelerated and leads to the oxidative stress response (AlSaad A. M. et al., 2020). A large amount of cholesterol or LDL accumulates in the blood and is deposited under the vascular endothelium, which is usually accompanied by vascular endothelial cell apoptosis and macrophage-dominated inflammation (Ma and Feng, 2016). Endothelial cell apoptosis and dysfunction induces a series of pro-inflammatory cytokines, which further aggravate the inflammatory reaction (Hou et al., 2019; Rudnicka et al., 2021). Macrophages phagocytose lipids are deposited under endothelial cells to form foam cells, which undergo the oxidative phosphorylation response. Massive lipid deposition in macrophages leads mitochondrial dysfunction and accelerates ROS production. The ROS converts LDL into a large amount of oxidized low-density lipoprotein (ox-LDL), and ox-LDL can cause inflammation and oxidative stress (Liao et al., 2021), which forms a vicious cycle. Thus, we tested the indexes related to oxidative stress and inflammation. The oxidative stress indexes showed that PF reduced the liver ROS content and the serum and liver MDA content and increased the serum and liver anti-oxidant indicators, including SOD, CAT, and GSH. For inflammatory indicators, PF decreased CRP, IL-1β, IL-6, and TNF- α in the serum and liver. This indicated that PF could reduce oxidative stress and the inflammatory response in hypercholesterolemic rats. These results are consistent with the study conducted by Xie et al. (Xie et al., 2018).

Systematic oxidative stress and the inflammatory response impair liver lipid metabolism, leading to liver lipid deposition in hypercholesterolemia (Stefan et al., 2019). We observed the hepatic pathological morphology using H&E staining and oil red O staining. We also determined the hepatic lipid content. The results showed that PF improved the liver structure, reduced inflammatory cell infiltration, and reduced lipid deposition in the liver. We also found that PF treatment reduced serum ALT and AST activity and the liver index. This suggests that PF can improve liver lipid deposition and protect the liver in hypercholesterolemic rats.

An important reason for hepatic lipid deposition is the increase of *in situ* lipid synthesis in the liver. SREBP-1c and FAS are important regulatory factors for *in situ* lipid synthesis in the liver. SREBP-1c is mainly expressed in the liver, and FAS is its key downstream pathway. Studies have shown that, in diet-induced obese mice, the effect of anti-hepatic steatosis is achieved by inhibiting SREBP-1c activity (Mun et al., 2019). The correlation between the inhibition of the SREBP-1c/FAS pathway and the reduction of liver lipid synthesis has been fully affirmed (Jiao et al., 2018; Fang et al., 2019). In this study, PF treatment significantly inhibited the expression of SREBP-1c and FAS, and finally reduced liver lipid deposition.

AMPK is the energy receptor of the body, and it plays a key role in regulating lipid metabolism (Chen et al., 2017). As a potential therapeutic target for metabolic diseases, AMPK is of great significance in treating NAFLD (Carling, 2017; Day et al., 2017). Activation of the AMPK pathway can inhibit the SREBP-1c and FAS expression, and reduce liver lipids synthesis. A recent study reported that ROCK is an upstream component of AMPK signaling, and the hepatic ROCK/AMPK signaling cascade is a crucial determinant of hepatic lipid synthesis (Sousa-Lima et al., 2021). Studies have shown that ROCK inhibition can improve hepatic steatosis and inhibit hepatic lipid synthesis (Huang et al., 2018; Sousa-Lima et al., 2021). ROCK inhibition can also activate AMPK and inhibit the downstream SREBP-1c and FAS, further reducing hepatic lipid deposition (Tang et al., 2017; Huang et al., 2018). Additionally, metformin, the most widely used anti-diabetes drug, can reduce liver lipid accumulation by inactivating ROCK (Özdemİr and Ark, 2021), which activates downstream AMPK signal transduction. Moreover, the ROCK signaling pathway regulates multiple biological functions throughout the body (Priviero et al., 2010) and is closely related to oxidative stress and inflammation. Our previous studies showed that inhibiting hepatic ROCK could improve oxidative stress and the inflammatory response in hypercholesterolemia (Ma et al., 2011a; Ma et al., 2011c). In this study, we used the p-MYPT-1 as a marker for ROCK activity (Innico et al., 2021), and we showed that p-MYPT-1 is highly expressed in the liver of hypercholesterolemic rats, which indicates that hepatic ROCK pathway expression was increased. After PF treatment, we found that p-MYPT-1 expression was significantly reduced, which also showed that PF could reduce hepatic oxidative stress and the inflammatory response, and these results are consistent with our previous research. Future studies should address the regulation mechanism of PF on ROCK/AMPK signaling pathway and clarify the target of PF.

## Conclusion

In this study, we found that PF exerted anti-inflammation and anti-oxidation, improved lipid deposition, and hepato-protective effects in hypercholesterolemic rats. The specific mechanism may be implemented through the ROCK/AMPK/SREBP-1c/FAS pathway. These findings suggest that PF may be a candidate drug for hypercholesterolemia, and further in-depth study of its mechanism and clinical applications are required.

## Data Availability

The raw data supporting the conclusion of this article will be made available by the authors, without undue reservation.
